# The MRC1/CD68 Ratio Is Positively Associated with Adipose Tissue Lipogenesis and with Muscle Mitochondrial Gene Expression in Humans

**DOI:** 10.1371/journal.pone.0070810

**Published:** 2013-08-12

**Authors:** José María Moreno-Navarrete, Francisco Ortega, María Gómez-Serrano, Eva García-Santos, Wifredo Ricart, Francisco Tinahones, Geltrude Mingrone, Belén Peral, José Manuel Fernández-Real

**Affiliations:** 1 Service of Diabetes, Endocrinology and Nutrition, Institut d'Investigació Biomèdica de Girona (IdIBGi), CIBEROBN (CB06/03/010) and Instituto de Salud Carlos III (ISCIII), Girona, Spain; 2 Instituto de Investigaciones Biomédicas ‘Alberto Sols’ (IIB), Consejo Superior de Investigaciones Científicas (CSIC) and Universidad Autónoma de Madrid (UAM), Madrid, Spain; 3 Department of Endocrinology and Nutrition, Hospital Virgen de la Victoria de Málaga, CIBEROBN Fisiopatologia Obesidad y Nutricion (CB06/03/018), Instituto de Salud Carlos III, Málaga, Spain; 4 Institute of Internal Medicine, Catholic University of Rome, Rome, Italy; Wageningen University, Netherlands

## Abstract

**Background:**

Alternative macrophages (M2) express the cluster differentiation (CD) 206 (MCR1) at high levels. Decreased M2 in adipose tissue is known to be associated with obesity and inflammation-related metabolic disturbances. Here we aimed to investigate *MCR1* relative to CD68 (total macrophages) gene expression in association with adipogenic and mitochondrial genes, which were measured in human visceral [VWAT, n = 147] and subcutaneous adipose tissue [SWAT, n = 76] and in *rectus abdominis* muscle (n = 23). The effects of surgery-induced weight loss were also longitudinally evaluated (n = 6).

**Results:**

*MCR1* and *CD68* gene expression levels were similar in VWAT and SWAT. A higher proportion of CD206 relative to total CD68 was present in subjects with less body fat and lower fasting glucose concentrations. The ratio *MCR1/CD68*was positively associated with *IRS1*gene expression and with the expression of lipogenic genes such as *ACACA, FASN* and *THRSP*, even after adjusting for BMI. The ratio *MCR1/CD68* in SWAT increased significantly after the surgery-induced weight loss (+44.7%; p = 0.005) in parallel to the expression of adipogenic genes. In addition, SWAT *MCR1/CD68*ratio was significantly associated with muscle mitochondrial gene expression (*PPARGC1A, TFAM* and *MT-CO3*). AT CD206 was confirmed by immunohistochemistry to be specific of macrophages, especially abundant in crown-like structures.

**Conclusion:**

A decreased ratio *MCR1/CD68* is linked to adipose tissue and muscle mitochondrial dysfunction at least at the level of expression of adipogenic and mitochondrial genes.

## Introduction

Obesity is associated with increased macrophage accumulation in adipose tissue [1]. Expression analysis of macrophage and non-macrophage cell populations isolated from adipose tissue demonstrate that adipose tissue macrophages are responsible for most of the proinflammatory cytokines and might contribute to obesity-linked inflammatory and metabolic complications [1]–[4]. Macrophages can exist in a proinflammatory classical state activated by interferon-γ or lipopolysaccharide, known as M1, or in an anti-inflammatory alternative state activated by IL-13 or IL-4, known as M2 [5]. Adipose tissue macrophages (ATMs) are composed of cells expressing MCR1, which is a marker of M2-activated macrophages [6], [7]. Recently, several studies reported that M2 macrophages in human and mouse adipose tissue were associated with less adipose tissue inflammation and obesity-associated metabolic disturbances [8]–[10]. A recent study in mice showed that early stages of adipose tissue expansion are characterized by M2-polarized ATMs and that progressive lipid accumulation within ATMs promotes the M1 polarization, a macrophage phenotype associated with severe obesity and insulin resistance. This polarization was reversed with rosiglitazone treatment, which promotes redistribution of lipids towards adipocytes, improving the adipose tissue lipid storage capacity [11].

In recent years, the interaction between adipose and muscle tissue has been increasingly recognized to play an important role in body weight regulation. Muscle mitochondrial dysfunction, which has been defined as a reduction in muscle oxidative capacity and mitochondrial biogenesis, has been associated with obesity, insulin resistance and type 2 diabetes [12]–[16]. Reduced levels of muscle PPARGC1A, TFAM and MT-CO3 mRNA levels have been used to evaluate muscle mitochondrial dysfunction [12], [13], [16].

We are not aware of studies in humans aimed to search for alternative macrophages in association with adipogenic genes, which may be used as markers for adipose tissue function: the higher the adipogenic gene expression, the higher the lipid storage capacity, or with muscle mitochondrial dysfunction in obese subjects.

We investigated the association of MCR1 and CD68 (a widely used macrophage marker, present in all macrophages subpopulations, M1 and M2) with the expression of adipose tissue-adipogenic genes in both cross-sectional and longitudinal (surgery-induced weight loss) studies, and with muscle mitochondrial genes (such as PPARGC1A, TFAM and MT-CO3).

## Materials and Methods

### CD68 and CD206 expression in adipose tissue, stromal vascular fraction (SVF) and in isolated adipocytes

A group of 147 visceral and 76 subcutaneous adipose tissue samples from participants, who were recruited at the Endocrinology Department of the Hospital Virgen de la Victoria (Malaga, Spain) and at the Endocrinology Service of the Hospital Universitari Dr. Josep Trueta (Girona, Spain), were analyzed. Finally, in a subgroup of 23 participants, muscle tissues (*rectus abdominis* muscle) were also obtained. All subjects were of Caucasian origin and reported that their body weight had been stable for at least three months before the study. Liver and renal diseases were specifically excluded by biochemical work-up, which consisted of specific liver and kidney functional tests. All subjects gave written informed consent, validated and approved by the ethical committee of the Hospital Universitari Dr. Josep Trueta and of the Hospital Virgen de la Victoria (Comitè d'Ètica d'Investigació Clínica, CEIC), after the purpose of the study was explained to them. Both the ethical committee of the Hospital Universitari Dr. Josep Trueta and the ethical committee of the Hospital Virgen de la Victoria specifically approved this study (ethical approval number 2008004). Adipose tissue samples were obtained from subcutaneous and visceral depots during elective surgical procedures (cholecystectomy, surgery of abdominal hernia and gastric by-pass surgery). Both subcutaneous and visceral fat were obtained from the abdomen, following standard procedures. To analyse adipose tissue and muscle gene expression, tissues were washed, fragmented and immediately flash-frozen in liquid nitrogen before stored at −80°C.

To perform the isolation of adipocyte and SVF, tissues were washed three to four times with phosphate-buffered saline (PBS) and suspended in an equal volume of PBS supplemented with 1% penicillin-streptomicin and 0.1% collagenase type I prewarmed to 37°C. The tissue was placed in a shaking water bath at 37°C with continuous agitation for 60 minutes and centrifuged for 5 minutes at 300 to 500 g at room temperature. The supernatant, containing mature adipocytes, was recollected. The pellet was identified as the SVF cell. The adipose tissue fractionation was performed from 12 visceral and 10 subcutaneous depots.

### Study of the effects of weight loss induced by bariatric surgery

Six Caucasian morbidly obese (BMI = 50.4±9.0 kg/m^2^, age = 40±10 years [mean ± SD]) women with normal glucose metabolism were recruited. The nature and purpose of the study were carefully explained to all subjects before they provided their written consent to participate.

At the time of the baseline study, all subjects were consuming a diet with the following average composition: 60% carbohydrate, 30% fat, and 10% protein (∼1 g/kg body weight). This dietary regimen was maintained for 1 week before the study. Patients underwent a clinical assessment including medical history, physical examination, body composition analysis, and co-morbidity evaluation, as well as nutritional interviews performed by a multidisciplinary consultation team. An oral glucose tolerance test (OGTT), an intravenous glucose tolerance test (IVGTT), and a euglycemic hyperinsulinemic clamp (EHC) were randomly performed within 1 month before surgery and 1 month after surgery. All patients received the same parenteral nutrition regimen (∼7,100 kJ/day) during the first 6 days after surgery; then they were free to consume a normal diet. All subjects were non-smokers and were not receiving statins or antidiabetic medication. Patients with signs of infection were excluded.

The malabsorptive surgical procedure consisted of a ∼60% distal gastric resection with stapled closure of the duodenal stump. The residual volume of the stomach is about 300 ml. The small bowel is transected at 2.5 m from the ileocecal valve, and its distal end is anastomosed to the remaining stomach. The proximal end of the ileum, comprising the remaining small bowel (involved in carrying biliopancreatic juice but excluded from food transit), is anastomosed in an end-to-side fashion to the bowel, 50 cm proximal to the ileocecal valve. Consequently, the total length of absorbing bowel is reduced to 250 cm, the final 50 cm of which, the so-called common channel, represents the site where ingested food and biliopancreatic juices mix.

The malabsorptive surgical procedure was performed as previously described [17] and subcutaneous adipose tissue samples and metabolic studies were again performed 2 years later. All subjects gave written informed consent, validated and approved by the institutional ethics committee of the Catholic University of Rome, after the purpose of the study was explained to them. The institutional ethics committee of the Catholic University of Rome specifically approved this study.

### Study of gene expressions

RNA was prepared from these samples using RNeasy Lipid Tissue Mini Kit (*QIAgen, US*). The integrity of each RNA sample was checked by Agilent Bioanalyzer (*Agilent Technologies, Palo Alto, CA*). Total RNA was quantified by means of spectrophotometer (*GeneQuant, GE Health Care, Piscataway NJ*) reverse transcribed to cDNA using High Capacity cDNA Archive Kit (*Applied Biosystems, Darmstadt, Germany*) according to the manufacturer's protocol.

Gene expression was assessed by real time PCR using an ABI Prism 7000 Sequence Detection System (*Applied Biosystems, Darmstadt, Germany*), using TaqMan*®* and SybrGreen technology suitable for relative gene expression quantification.

The commercially available and pre-validated TaqMan® primer/probe sets used were as follows: endogenous control PPIA (4333763, cyclophilin A) and target genes CD68 (*CD68*, Hs00154355_m1), mannose receptor, C type 1 (*MRC1* or *CD206,* Hs00267207_m1), fatty acid synthase (*FASN*, Hs00188012_m1), acetyl-Coenzyme A carboxylase alpha (*ACC*, Hs00167385_m1), insulin receptor substrate 1 (*IRS1*, Hs00178563_m1), SPOT14 homolog (*THRSP,* Hs00930058_m1), leptin (*LEP*, Hs00174877_m1). Human PPARGC1A [forward: 5′- GCAATTGAAGAGCGCCGTGTGA-3′ and reverse: 5′- CTGTCTCCATCATCCCGCAGAT-3′], TFAM [forward: 5′- AAGATTCCAAGAAGCTAAGGGTGA-3′and reverse: 5′- CAGAGTCAGACAGATTTTTCCAGTTT-3′], MT-CO3 [forward: 5′- GCCCCCAACAGGCATCA-3′ and reverse: 5′- GGATGTGTTTAGGAGTGGGACTTC-3′] were measure using SYBRgreen technology.

The RT-PCR TaqMan® reaction was performed in a final volume of 25 μl. The cycle program consisted of an initial denaturing of 10 min at 95°C then 40 cycles of 15 sec denaturizing phase at 95°C and 1min annealing and extension phase at 60°C. A threshold cycle (Ct value) was obtained for each amplification curve and a ΔCt value was first calculated by subtracting the Ct value for human *Cyclophilin A* (PPIA) RNA from the Ct value for each sample. Fold changes compared with the endogenous control were then determined by calculating 2^−ΔCt^, so gene expression results are expressed as expression ratio relative to PPIA gene expression according to manufacturers' guidelines.

### Immunofluorescence

Five-micron sections of formalin-fixed paraffin-embedded adipose tissue were deparaffinised and rehydrated prior to antigen unmasking by boiling in 1 mM EDTA, pH 8. Sections were blocked in normal serum and incubated overnight with mouse anti CD206 antibody at 1∶50 dilution, and rabbit anti CD68 at 1∶50 dilution, washed, and visualized using Alexa Fluor 488 goat anti-mouse antibody and Alexa Fluor 546 goat anti-rabbit (1∶500; Molecular Probes Inc, OR, USA), respectively. As a negative control, the entire immunofluorescence procedure was performed in the absence of primary antibody. The slides were counterstained with DAPI (4,6-diamidino-2-phenylindole) to reveal nuclei and examined under a Leica TCS SP5 fluorescent microscope (Heidelberg, Germany).

### Statistical analyses

Statistical analyses were performed using SPSS 12.0 software. Unless otherwise stated, descriptive results of continuous variables are expressed as mean and SD for Gaussian variables, or median and interquartile range. Parameters that did not fulfill normal distribution were mathematically transformed to improve symmetry for subsequent analyses. The relation between variables was analyzed by simple correlation (Spearman's test). One-way ANOVA (using Bonferroni Post hoc test) was used to compare clinical variables and *CD206* and *CD68* gene expressions according to obesity status and adipose tissue fractions. Statistical significance was set at P<0.05.

## Results

**Figure 1:**
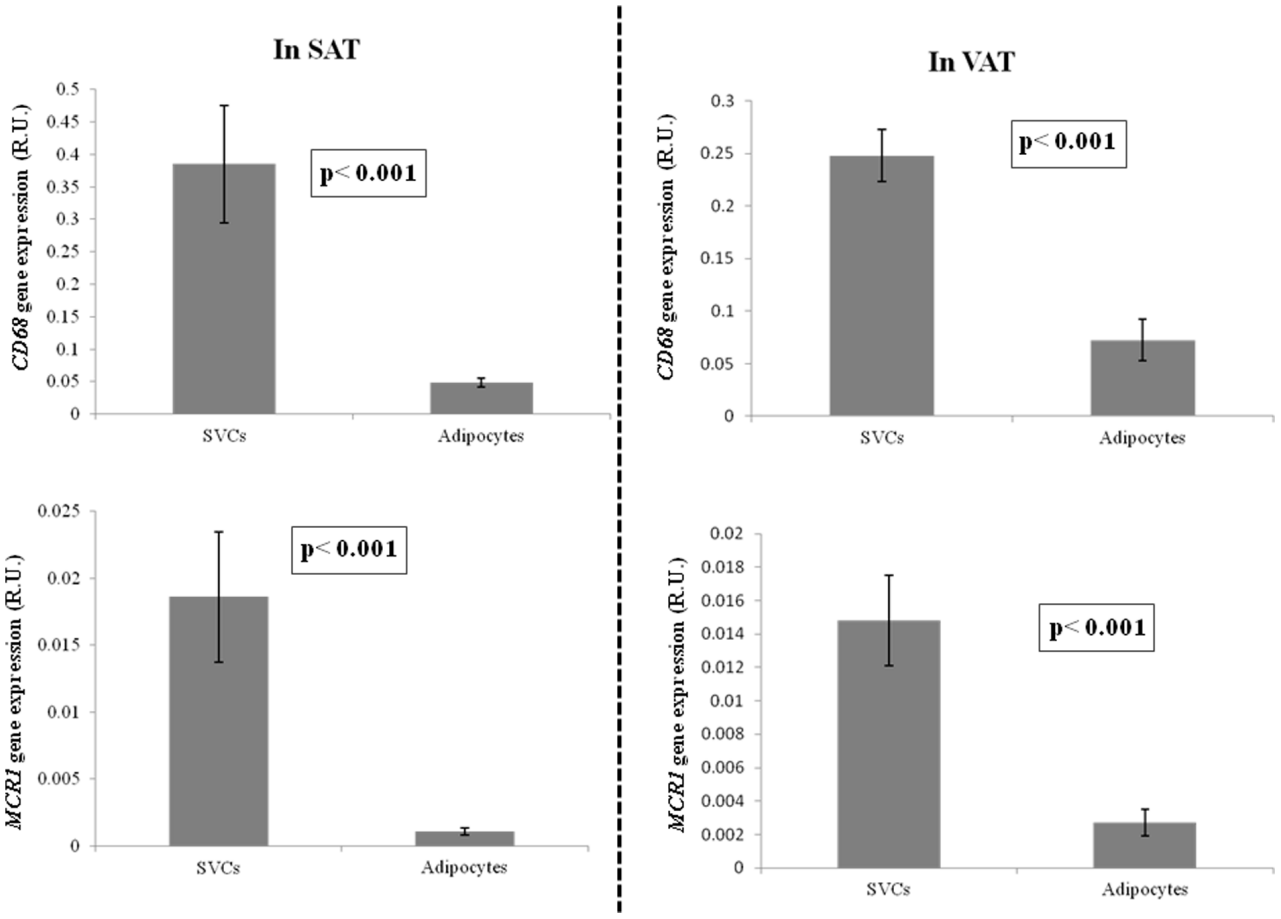
*CD68* and *MCR1* gene expression in subcutaneous (n = 10) and visceral (n = 12) adipose tissue fractions (SVCs and adipocytes).

**Figure 2:**
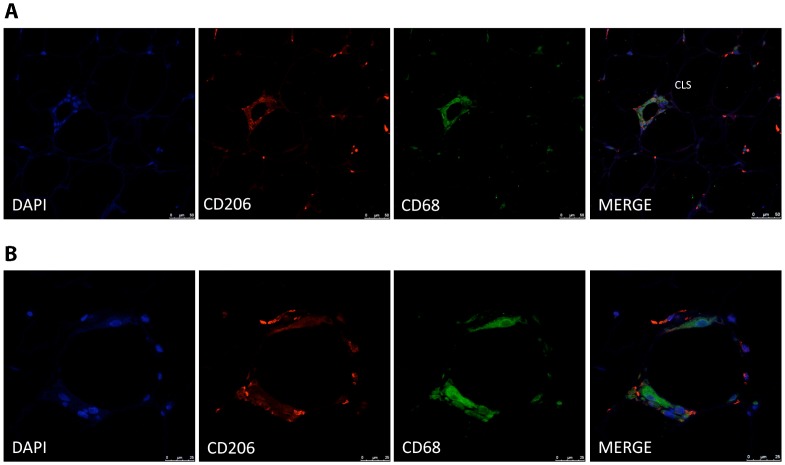
Immunofluorescence detection of CD206 and CD68 in human adipose tissue crown-like structures. Co-staining of adipose tissue macrophages in “crown-like structures” demonstrates co-localization of CD206 (red) and CD68 (green) proteins in most of the cells (A). Close-up view of a CLS (B) The counterstaining of nuclei (DAPI) is shown in blue. Images are representative of adipose tissue preparations collected from three subjects. Scale bar, 50 µm (A) and 25 µm (B).

As expected, *CD68* and *MCR1* gene expression were significantly increased in stromal vascular cell fraction (SVF) in comparison with adipocytes (Figure 1). Immunostaining analyses were performed to determine the cellular distribution of CD206 protein adipose tissue. Immunofluorescence showed a bright staining pattern in cells resembling adipose tissue macrophages. In fact, CD206 stained cells were well represented in inflammatory crown like structures (CLS) (Figure 2). Co-staining analysis using CD206 and CD68 antibodies showed that most of the CD206 cells were also stained with CD68 antibody. However a few cells remained stained only for CD206 in the CLS.

Anthropometrical and clinical data from the participants are detailed in Table 1. The levels of gene expression of *CD68, MCR1* and *MCR1/CD68* were similar in both visceral and subcutaneous adipose tissue. In visceral adipose tissue, *MCR1/CD68* ratio was reduced in obese subjects, mainly in those with type 2 diabetes, whereas *CD68* gene expression was increased. *MCR1* gene expression did not change according to obesity or type 2 diabetes (Table 1).

**Table 1:** Anthropometrical and clinical parameters.

	Non- obese	Obese	Obese T2DM	p
**N**	59	56	31	
**Sex**	23/36	10/46	8/23	
**Age (years)**	49.1±14.8	46.5±13.6	46.3±11.7	0.5
**BMI (Kg/m^2^)**	24.7±2.9	44.1±8.6*	40.9±8.03*	**<0.0001**
**Fat mass (%)**	31.37±*7.1*	*56.44*±11.2*	51.6±11.4*	**<0.0001**
**Fasting glucose (mg/dl)**	83.2±10.4	95.9±13.8*	136.6±55.8* ^#^	**<0.0001**
**Fasting Insulin (µU/ml)**	12.11±6.1	14.09±7.1	18.35±8.2*	**0.01**
**HOMA-IR**	2.49±1.2	3.5±1.9	5.8±2.3* ^#^	**<0.0001**
**In VWAT**				
**IRS1** +	0.017±0.011	0.01±0.005	0.009±0.004	**<0.0001**
**LEP** +	0.15±0.12	0.29±0.14*	0.40±0.21*	**<0.0001**
**THRSP** +	0.55±0.4	0.18±0.1*	0.11±0.07*	**<0.0001**
**FASN** +	0.27±0.12	0.076±0.060*	0.061±0.050*	**<0.0001**
**ACC1** +	0.032±0.02	0.016±0.010*	0.014±0.011*	**<0.0001**
**CD68** +	0.12±0.07	0.18±0.07*	0.23±0.1*	**<0.0001**
**CD206** +	0.022±0.018	0.021±0.013	0.021±0.010	0.8
**CD206/ CD68** +	0.18±0.08	0.12±0.08*	0.11±0.07*	**<0.0001**
**In SWAT**				
**IRS1** +	0.010±0.005	0.011±0.006	0.009±0.003	0.2
**LEP** +	1.09±0.3	0.97±0.3	0.81±0.26	0.2
**THRSP** +	0.46±0.1	0.38±0.2	0.37±0.2	0.5
**FASN** +	0.25±0.20	0.06±0.04*	0.05±0.03*	**<0.0001**
**ACC1** +	0.019±0.009	0.021±0.01	0.016±0.006	0.3
**CD68** +	0.17±0.05	0.2±0.1	0.22±0.09	0.5
**CD206** +	0.013±0.005	0.016±0.01	0.018±0.009	0.3
**CD206** + **/ CD68** +	0.076±0.018	0.084±0.03	0.088±0.028	0.6

+ Relative gene expression (R.U.).

The data are expressed as mean ± standard deviation.

VWAT, visceral white adipose tissue; SWAT, subcutaneous white adipose tissue; T2DM, Type 2 Diabetes Mellitus; HOMA, Homeostasis Model Assessment; R.U., relative gene expression units.

* p<0.05 compared with non-obese participants, performing Bonferroni post hoc test.

A higher proportion of MCR1 relative to total CD68 macrophages was present in subjects with less body fat [BMI (r = −0.44, p<0.0001), fat mass (r = −0.44, p<0.0001)] and less fasting glucose concentrations (r = −0.17, p = 0.04). Interestingly, the ratio *MCR1/CD68* was positively associated with *IRS1* (r = 0.4, p<0.0001) and *ADIPOQ* gene expression (r = 0.4, p<0.0001) and with expression measures for lipogenic genes such as *ACACA* (r = 0.36, p<0.0001), *FASN* (r = 0.44, p<0.0001) and *THRSP* (r = 0.43, p<0.0001). In multiple linear regression models, *IRS1* (β = 0.24 p = 0.003, R^2^ = 0.17), *ADIPOQ* (β = 0.77 p<0.0001, R^2^ = 0.55), *THRSP* (β = 0.20 p = 0.04, R^2^ = 0.15), *FASN* (β = 0.19 p = 0.045, R^2^ = 0.13) but not *ACACA* (β = 0.15 p = 0.06, R^2^ = 0.10) gene expression contributed independently to *MCR1/CD68* gene expression variance after adjusting for BMI. Oppositely, *CD68* gene expression was directly correlated with BMI (r = 0.45, p<0.0001), fat mass (r = 0.41, p<0.0001), fasting glucose (r = 0.18, p = 0.03), and with the gene expression of *LEP* (r = 0.27, p = 0.009), and negatively correlated with *IRS1* (r = −0.17, p = 0.04), *THRSP* (r = −0.29, p = 0.002), *ACACA* (r = −0.24, p = 0.01), *FASN* (r = −0.31, p<0.0001). These associations were lost after controlling for BMI. No associations were observed among *CD68, MCR1/CD68* and fasting insulin or HOMA-IR values.

In subcutaneous adipose tissue (SWAT), *MCR1/CD68* and *CD68* were not significantly associated with obesity or type 2 diabetes (Table 1). The ratio *MCR1/CD68* was positively associated with *ADIPOQ* gene expression (r = 0.36, p<0.0001).

**Figure 3:**
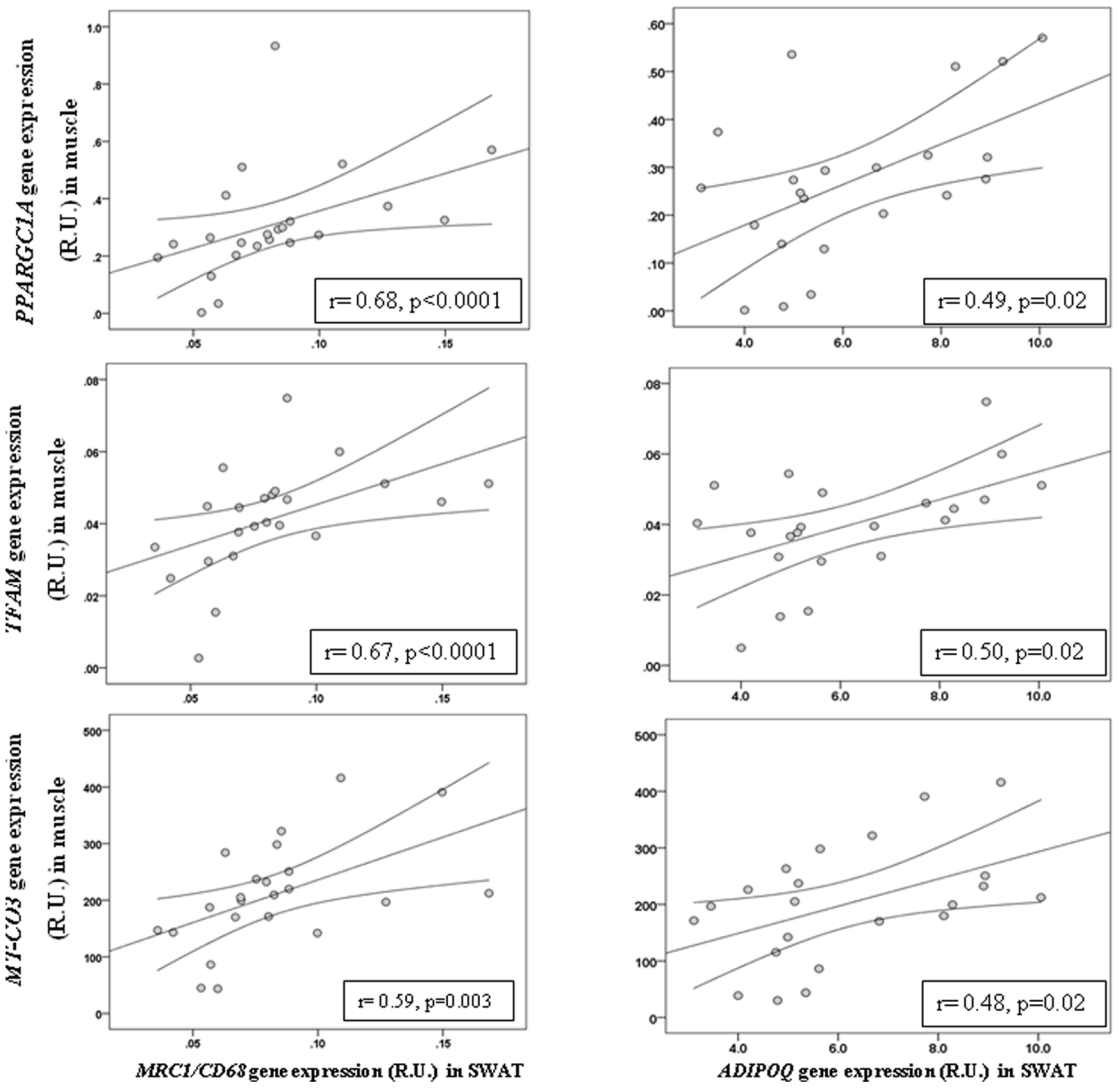
Correlation between SWAT *MCR1/CD68* and *ADIPOQ* and muscle *PPARGC1A, TFAM* and *MT-CO3* gene expression.

Interestingly, in a subcohort of 23 participants, where muscle biopsies were available, SWAT *MCR1/CD68* ratio and *ADIPOQ* gene expression was significantly associated with the gene expression of mitochondrial biogenesis and activity in muscle (*PPARGC1A, TFAM* and *MT-CO3*, Figure 3).

### Weight loss-induced effects

**Figure 4:**
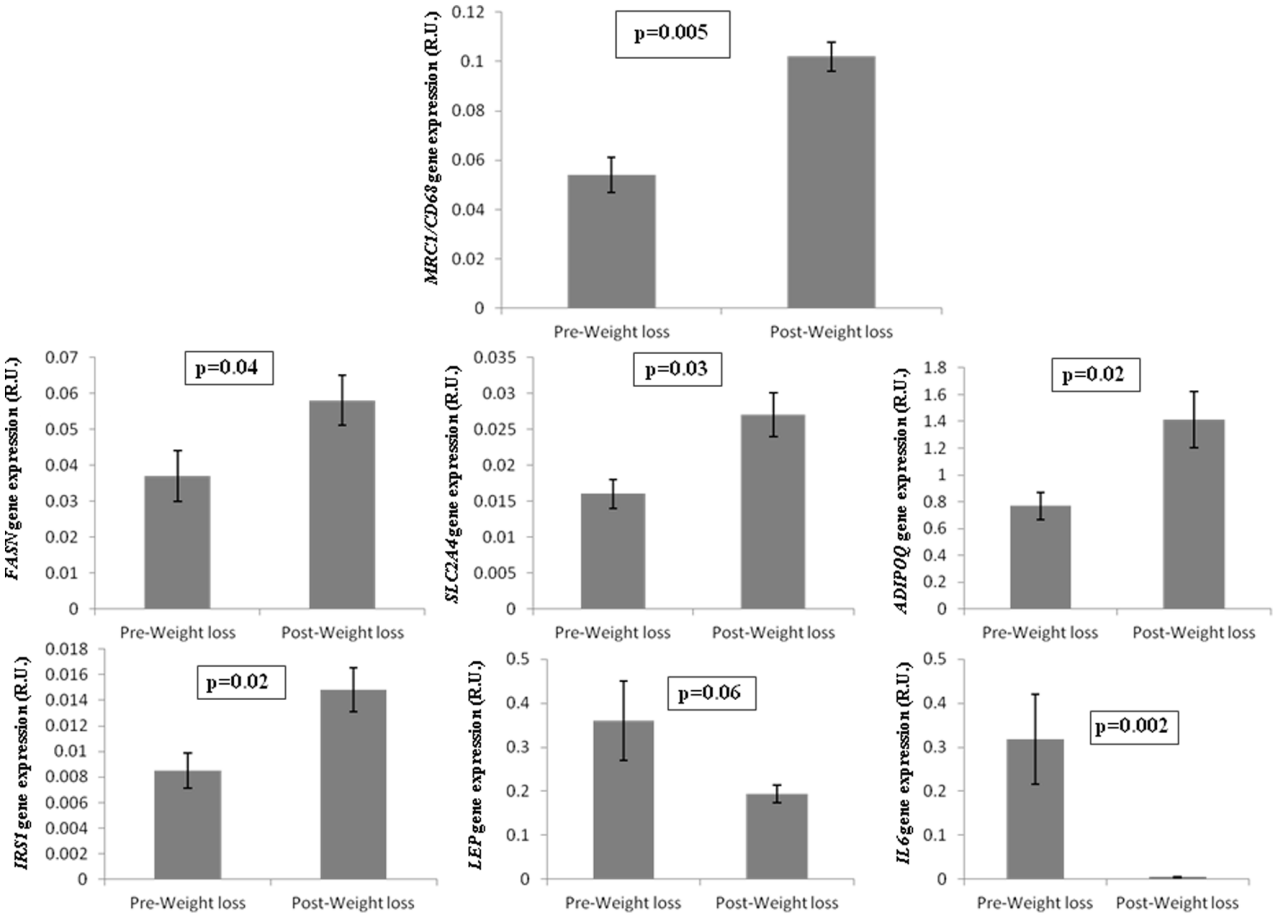
Effects of bariatric surgery-induced weight loss in adipose tissue *MCR1/CD68*, *FASN, ADIPOQ, IRS1*, *SLC2A4*, *LEP* and *IL6* gene expression.

To study the effects of fat mass reduction on the ratio *MCR1/CD68* gene expression in adipose tissue, they were studied in subcutaneous adipose tissue at baseline and after bariatric surgery-induced weight loss. *MCR1/CD68* ratio increased significantly after weight loss (Figure 4) in parallel to adipogenic genes, such as *FASN, ADIPOQ, IRS1* and *SLC2A4*, whereas *LEP* (an obesity gene marker) and *IL6* (an inflammatory marker) decreased (Figure 4).

## Discussion

PPARγ activation is known to promote infiltration of alternatively activated macrophages into mice adipose tissue, increasing the proportion of CD206^+^ cells. In fact, Stienstra et al. proposed that the rosiglitazone-induced increase in CD206^+^ cells is one of the mechanisms which contribute to the beneficial effects of rosiglitazone in parallel to adipose tissue expandability [18]. On the other hand, it is well-known that the gene expression of adipogenic genes (both lipogenic and insulin signaling-related genes) is positively linked to adipose tissue expandability (lipid storage capacity) and to protection from developing obesity-related metabolic disturbances [19]–[21].

One of the main findings of the present study may suggest a possible communication between tissues. SWAT *MCR1/CD68* ratio was strongly associated with the muscle gene expression of mitochondrial genes (*PPARGC1A, TFAM* and *MT-CO3*). As both muscle and fat were sampled from the same abdominal territory (fat in contact with the *rectus abdominis* muscle), this finding led us to speculate a possible functional link between healthy adipose tissue expansion and muscle mitochondrial activity, known to be impaired in obesity-associated metabolic disturbances [22]. Both adipose and muscle tissues secrete cytokines and other peptides, named adipokines and myokines, which contribute to tissue communication that is essential to maintain metabolic homeostasis. For instance, adiponectin is an hormone produced specifically by adipose tissue which increases AMPK-induced oxidative metabolism and glucose uptake by the muscle [23], [24]. The strong association between *ADIPOQ* gene expression and the MRC1/CD68 ratio led us to suggest that circulating adiponectin might underlie in the positive connection between MRC1/CD68 and muscle mitochondrial gene expression. Supporting this hypothesis the positive effects of adiponectin increasing muscle fat oxidation and mitochondrial biogenesis have been shown in an extensive number of studies [23]–[28].The measurement of circulating LMW-adiponectin concentration might be a possible limitation of this study, since serum or plasma samples from this cohort were not available. However, it is well-known that adiponectin biosynthesis is exclusively produced in adipose tissue. In a recent study, epicardial adipose tissue volume has been positively associated with CD68 and inflammatory cytokines, and negatively with adiponectin gene expression, being these associations strongly linked to human coronary atherosclerosis [29]. This study supports the possible role of adiponectin connecting adipose tissue and muscle functionality. Otherwise, some myokines such as IL6, IL15 and irisin display beneficial effects on metabolism interacting with the adipose tissue [30]. Exercise-induced muscle IL6 production increases insulin-stimulated glucose metabolism in muscle and adipose tissue, enhancing insulin-stimulated glucose disposal and fatty acid via AMPK activation [31]. IL15 leads to reduced adipogenesis and increased fatty acid mobilization from adipose tissue depots [32], [33]. Recently, irisin has been proposed to mediate the beneficial effects of exercise on metabolism, inducing browning of subcutaneous adipocytes and increasing total body energy expenditure and resistance to obesity-associated insulin resistance [34], [35].”

We here also describe a positive association between the *MRC*1*/CD68* gene expression ratio and lipogenic gene expression in both cross-sectional and longitudinal studies. To the best of our knowledge, this finding is novel in human adipose tissue. The *MRC1/CD68* gene expression ratio also decreased progressively with increased fatness. The presence of CD206^+^ macrophages in human adipose tissue is known to be associated with a healthy adipose tissue expansion, i.e. an enlargement of adipose tissue through effective recruitment of adipogenic precursor cells to the adipogenic program in parallel to an adequate angiogenic response and appropriate remodeling of the extracellular matrix [36]. In this context, CD206^+^ macrophages are inversely associated with metabolic disturbances [10], [37]. A small subpopulation of CD206^+^CD11^+^ (less than 10% of total CD206^+^, which are CD11^−^) was also significantly associated with obesity and insulin resistance [37].

It is extensively well shown that the number of macrophages in adipose tissue increases with obesity-associated inflammation [1], [4]. Here, we confirmed an increased *CD68* gene expression in association with obesity. In fact, the associations of *CD68* gene expression with fasting glucose, leptin and adipogenic gene expression were closely dependent on obesity. Bariatric surgery-induced weight loss led to increased *MCR1/CD68* ratio in parallel to the adipogenic status of SWAT, confirming a previous immunohistochemistry study [9].

The most significant associations of both *MCR1/CD68* and *CD68* levels with obesity in the cross-sectional study were mainly found in visceral adipose tissue. Visceral adipose tissue is a well known contributor to insulin resistance [38], [39]. In fact, both lipogenic and *IRS1* gene expression are known to be significantly decreased in visceral adipose tissue [40], [41].

In conclusion, a decreased *MCR1/CD68* ratio (mainly in visceral adipose tissue) is a potential marker of obesity-associated adipose tissue dysfunction, and linked to decreased expression of mitochondrial genes in the muscle.
